# A Case Control Association Study and Cognitive Function Analysis of Neuropilin and Tolloid-Like 1 Gene and Schizophrenia in the Japanese Population

**DOI:** 10.1371/journal.pone.0028929

**Published:** 2011-12-20

**Authors:** Masahiro Banno, Takayoshi Koide, Branko Aleksic, Kazuo Yamada, Tsutomu Kikuchi, Kunihiro Kohmura, Yasunori Adachi, Naoko Kawano, Itaru Kushima, Masashi Ikeda, Toshiya Inada, Takeo Yoshikawa, Nakao Iwata, Norio Ozaki

**Affiliations:** 1 Department of Psychiatry, Nagoya University Graduate School of Medicine, Showa-ku, Nagoya, Aichi-ken, Japan; 2 Department of Psychiatry, Fujita Health University School of Medicine, Kutsukake, Toyoake, Aichi-ken, Japan; 3 Laboratory for Molecular Psychiatry, RIKEN Brain Science Institute, Wako-city, Saitama, Japan; 4 Matsuzaki Hospital, Miyakojima-ku, Osaka, Japan; 5 Department of Psychiatry, Seiwa Hospital, Institute of Neuropsychiatry, Shinjuku-ku, Tokyo, Japan; Central China Normal University, China

## Abstract

**Background:**

Using a knock-out mouse model, it was shown that NETO1 is a critical component of the NMDAR complex, and that loss of *Neto1* leads to impaired hippocampal long term potentiation and hippocampal-dependent learning and memory. Moreover, hemizygosity of *NETO1* was shown to be associated with autistic-like behavior in humans.

**Purpose of the Research:**

We examined the association between schizophrenia and the neuropilin and tolloid-like 1 gene (*NETO1*). First, we selected eight single nucleotide polymorphisms (SNPs) within the *NETO1* locus, based on the Japanese schizophrenia genome wide association study (JGWAS) results and previously conducted association studies. These SNPs were genotyped in the replication sample comprised of 963 schizophrenic patients and 919 healthy controls. We also examined the effect of associated SNPs on scores in the Continuous Performance Test and the Wisconsin Card Sorting Test Keio version (schizophrenic patients 107, healthy controls 104).

**Results:**

There were no significant allele-wise and haplotype-wise associations in the replication analysis after Bonferroni correction. However, in meta-analysis (JGWAS and replication dataset) three association signals were observed (rs17795324: p = 0.028, rs8098760: p = 0.017, rs17086492: p = 0.003). These SNPs were followed up but we could not detect the allele-specific effect on cognitive performance measured by the Continuous performance test (CPT) and Wisconsin Card Sorting test (WCST).

**Major Conclusions:**

We did not detect evidence for the association of *NETO1* with schizophrenia in the Japanese population. Common variants within the *NETO1* locus may not increase the genetic risk for schizophrenia in the Japanese population. Additionally, common variants investigated in the current study did not affect cognitive performance, as measured by the CPT and WCST.

## Introduction

The glutamate hypothesis of schizophrenia (GHS) emerged in the early 1980 s as an alternative to the prevailing theory of altered dopamine neurotransmission. The GHS is based on the observation that non-competitive antagonists of the N-methyl-d-asparate (NMDA) subtype of glutamate receptors, such as phencyclidine (PCP), ketamine and MK-801, induce a psychotic reaction in healthy individuals that resembles schizophrenia (both the positive and negative symptoms). When the same compounds are administered to patients with schizophrenia, exacerbation of psychotic symptoms can be the outcome [1]. Together, these observations suggest that diminished function of the NMDA receptor (NMDAR) may play a role in the pathoetiology of schizophrenia. Moreover, evidence from morphological, clinical and neuroimaging studies have provided support for the GHS by mapping cognitive impairment, alterations in blood flow and changes in neuronal morphology to particular brain areas, including the frontal and cingulate cortices, both of which are areas with extensive excitatory glutamatergic neurotransmission [2].

**Figure 1:**
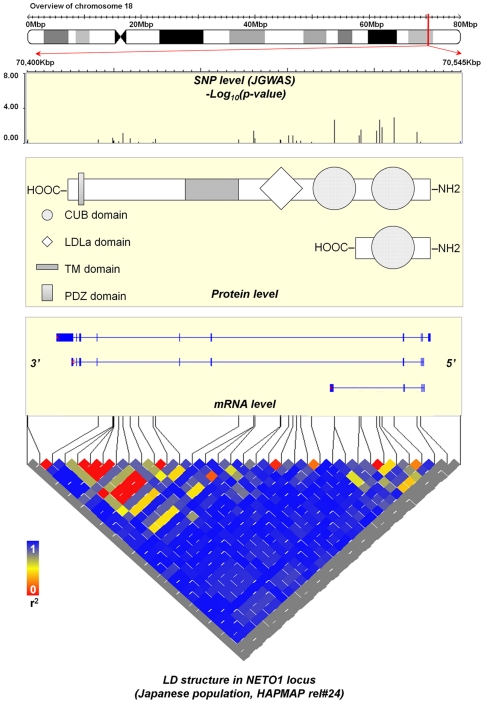
NETO1-gene centric data. LD: linkage disequilibrium. NETO1 maps to the 18q22-q23 and three alternative splicing variants (mRNA level) have been observed. Specifically variants 1 and 2 are detected in retina while variant 3 is specific for fetal and adult brain (mRNA level panel). NETO1 is a transmembrane protein, which has two extracellular CUB domains, a low-density lipoprotein class A (LDLa) domain, a transmembrane domain and a classical type I PDZ-domain binding motif (protein level panel).

The N-methyl-D-aspartate receptor (NMDAR), a major excitatory ligand-gated ion channel in the central nervous system, is composed of a heterotetramer between two NR1 and two NR2 subunits. Moreover, the NMDAR is a principal mediator of synaptic plasticity [3]. It has been shown that corticolimbic NMDAR hypofunction is one of the core molecular mechanisms relevant for phenotypes observed in animal models of schizophrenia [4]. One of the genes that regulate NMDAR function is neuropilin and tolloid-like 1 gene (*NETO1*). *NETO1* maps to the 18q22-q23 and three alternative splicing variants (mRNA level) have been observed [5]. Specifically, variants 1 and 2 are detected in the retina while variant 3 is specific for fetal and adult brain. NETO1 is a transmembrane protein, which has two extracellular CUB domains, a low-density lipoprotein class A (LDLa) domain, a transmembrane domain and classical type I PDZ-domain binding motif [5] (Figure 1). Deletion of *Neto1* leads to deficits in synaptic plasticity in mice while stimulation of the AMPA receptor can partially compensate for deficits caused by *Neto1* deletion [6]. NETO1 interacts with the core NMDAR subunits, NR2A and NR2B and a scaffolding protein, postsynaptic density-95 (PSD-95), maintaining the abundance of NR2A-containing NMDARs in the postsynaptic density of the hippocampus. PSD-95 is a protein that is almost exclusively located in the postsynaptic density of neurons, and is important in anchoring synaptic proteins [7]. Increase in surface NR2A, but not NR2B, occurs in hippocampal neurons derived from dysbindin-null mutant mice (Dys-/-). Dysbindin controls hippocampal LTP by selective regulation of the surface expression of NR2A [8]. In situ hybridization studies of schizophrenia detected decreased transcript expression of the NR1 subunit, increased transcript expression of the NR2B subunit and unchanged transcript expression of the NR2A subunit in hippocampus [9]. Therefore, regulation of NR2 in hippocampus in schizophrenia may be relevant for the etiology of schizophrenia and NETO1 may play an important role in the molecular mechanism by maintaining the abundance of NR2A-containing NMDARs in the postsynaptic density of hippocampal neurons. Moreover, NETO1 interacts with kainate receptors (KAR), one of the glutamate receptors, in mouse brain. NETO1 modulates the KAR affinity for the endogenous ligand glutamate. NETO1 modulates not only kinetics, but also the amplitude of slow excitatory postsynaptic current in KAR (KAR-EPSC) [10], [11]. NETO1 fundamentally alters the function and neuronal localization of GluK1-containing KAR [10], [11]. Therefore, NETO1 may influence glutamate neurotransmission through modulation of KAR and NMDAR properties.

Genetic studies suggested that the *NETO1* locus could harbor genetic variants that are relevant for susceptibility to neuropsychiatric disorders. Specifically, hemizygosity of *NETO1* was shown to be associated with a autistic-like behaviors in humans [12]. Although unequivocal genome wide evidence (p<10^−8^) for association at the *NETO1* locus has not been reported, it is of note that in the recent GWASs of Alzheimer's disease and schizophrenia, suggestive association signals were observed (rs1109070; p = 0.000669 [13] and rs9962470; p = 0.000154 [14] in Alzheimer's disease and schizophrenia, respectively). Moreover, several weak association signals (P<0.05) within the *NETO1* locus were detected in the first GWAS of schizophrenia conducted in the Japanese population (JGWAS) [15]. It is of note that in the JGWAS, genome wide evidence for association was not detected, and the non-genome wide level of statistical significance should be interpreted with caution. However due to the relatively small sample size, type II errors (false negative result) cannot be excluded – especially in the case of small odds ratios (OR) which are expected for common SNPs associated with schizophrenia [16].

Based on the aforementioned biological studies, *NETO1* can be seen as a promising candidate gene for schizophrenia. However, to the best of our knowledge, no genetic association study specifically designed to evaluate the association between *NETO1* and schizophrenia has been conducted. The goal of the present study was to evaluate the association between *NETO1* and schizophrenia based on the JGWAS. Additionally, as deficits were found in LTP and learning and memory in *Neto1*-null mice [7], we performed a cognitive function analysis that targeted the relationship between common SNPs selected based on the JGWAS and cognitive function assessed by the CPT and the WCST.

## Results

In the replication sample set, we did not detect any association between eight SNPs and schizophrenia after Bonferroni correction was applied (Table 1). Using the replication sample set, we have conducted haplotype-wise analysis in order to investigate association between haplotypes within the *NETO1* locus and schizophrenia. We did not detect evidence for haplotype-wise association after Bonferroni correction was applied (Table S1). In order to maximize the power, we performed meta-analysis combining results from the JGWAS and the replication dataset. In this analysis we could detect three suggestive association signals (rs17795324: P = 0.028, rs8098760: P = 0.017, rs17086492: P = 0.003). In the test of heterogeneity, we detected four SNPs, which showed significant high heterogeneities (Breslow-Day test; rs17795324: P = 0.04, rs6566674: P = 0.01, rs8098624: P = 0.003, rs1109070: P = 0.0001) (Table 2). However, we could not detect any association between these three SNPs (rs17795324, rs8098760 and rs17086492) and CPT and WCST scores (Table S4). We investigated the association between the *NETO1* gene and schizophrenia stratified by gender. We could detect no association signals either for males or females in the meta-analysis after Bonferroni correction (Tables S2 and S3).

**Table 1:** Results of JGWAS (N = 1108).

SNP	Position^a^	Minor allele	Case^b^	Control^b^	p-value^c^	OR	L95^d^	U95^d^
rs9962470	68616086	A	0.03	0.04	0.31	0.79	0.50	1.24
rs17086334	68626752	C	0.10	0.08	**0.05**	1.34	1.00	1.80
rs17795324	68654033	G	0.27	0.33	**0.003**	0.76	0.63	0.91
rs6566674	68662791	T	0.20	0.16	**0.04**	1.26	1.01	1.57
rs8098624	68669199	T	0.30	0.24	**0.002**	1.35	1.12	1.63
rs8098760	68669970	T	0.11	0.08	**0.02**	1.42	1.06	1.90
rs17086492	68674050	C	0.14	0.10	**0.001**	1.52	1.17	1.98
rs1109070	68674559	C	0.21	0.16	**0.01**	1.35	1.09	1.68

a based on NCBI 36.

b minor allele frequency.

c Fisher's exact test.

d Lower (L) and upper (U) 95% confidence intervals.

**Table 2:** Allele frequencies of the eight SNPs of *NETO1*.

SNP	Position^a^	Minor allele	Replication (N = 1882)						Meta analysis (N = 2990)				
			Case^b^	Control^b^	p-value^c^	OR	L95^d^	U95^d^	p-value^c^	OR	L95^d^	U95^d^	BDp^e^
rs9962470	68616086	A	0.02	0.03	0.24	0.79	0.53	1.18	0.126	0.79	0.59	1.07	0.99
rs17086334	68626752	C	0.08	0.08	0.87	0.98	0.77	1.25	0.289	1.11	0.92	1.33	0.09
rs17795324	68654033	G	0.29	0.30	0.61	0.96	0.84	1.11	**0.028**	0.88	0.79	0.99	0.04
rs6566674	68662791	T	0.17	0.19	0.12	0.88	0.74	1.04	0.926	1.01	0.88	1.15	0.01
rs8098624	68669199	T	0.27	0.29	0.43	0.94	0.82	1.09	0.213	1.08	0.96	1.21	0.003
rs8098760	68669970	T	0.10	0.08	0.18	1.17	0.93	1.47	**0.017**	1.24	1.04	1.49	0.26
rs17086492	68674050	C	0.12	0.11	0.22	1.14	0.93	1.39	**0.003**	1.28	1.09	1.50	0.09
rs1109070	68674559	C	0.16	0.19	**0.01**	0.78	0.65	0.93	0.718	0.98	0.85	1.12	0.0001

a based on NCBI 36.

b minor allele frequency.

c Fisher's exact test.

d Lower (L) and upper (U) 95% confidence intervals.

e p-value of Breslow-Day test.

## Discussion

In this study, we investigated the association between eight SNPs within *NETO1* and schizophrenia in the Japanese population. We detected associations between *NETO1* and schizophrenia in the meta-analysis, however, as the JGWAS dataset was included in the meta-analysis, evidence for association might be overestimated. In order to address this issue we tested the association between candidate SNPs from our meta-analysis and cognitive performance measured by the CPT and WCST. This analysis was of interest for us as *Neto1*-null mice show deficits in LTP and learning and memory [7], and if the three SNPs (rs17795324, rs8098760, rs17086492) were genuinely associated with schizophrenia, then carriers of risk alleles would likely have deficits in cognitive processing assessed by CPT and WCST [6]. However, we could not detect any association between these three SNPs (rs17795324, rs8098760, rs17086492) and the psychological tests we applied. We also performed meta-analysis (Method S1) of rs6566674 (the SNP that was included both in our replication sample set and another Japanese GWAS [17]), however we did not detect evidence for an association with schizophrenia. These results suggest that *NETO1* is not associated with schizophrenia in the Japanese population.

We detected four SNPs, which showed heterogeneous association (Breslow-Day test; rs17795324: P = 0.04, rs6566674: P = 0.01, rs8098624: P = 0.003, rs1109070: P = 0.0001) in the meta-analysis. These high heterogeneities may be derived from flip-flop phenomenon, that is, associations of opposite alleles at the same biallelic locus with the same disease [18]. Although the flip-flop phenomenon may represent a genuine genetic association (i.e., genuinely different LD architectures across populations with different ancestral origins), it may also be an artifact due to sampling variation that leads to variability in observed LD patterns.

Several caveats should be considered when interpreting the results of our study. First, in terms of sample size, the replication dataset may not have sufficient statistical power to detect associations between SNPs with low genotype relative risk (GRR) and schizophrenia. In other words, our sample has statistical power greater than 0.8 for the detection of association signals at nominal statistical significance, of the polymorphism with a minor allele frequency of 0.1, when the GRR is 1.30. Therefore, the possibility of association between schizophrenia and common SNPs with GRR<1.30 cannot be excluded. Furthermore, the JGWAS may not have sufficient power to detect associations between SNPs with low GRR and schizophrenia. Therefore, other relevant common variants in the *NETO1* region, which the JGWAS cannot identify, may exist. In addition, association between *NETO1* and schizophrenia may be specific only for the patients with early onset age. However, as in the current study, the frequency of the early onset schizophrenia patients was low (less than 5%). The power to detect such an association is inadequate. Therefore, the effect of onset age on the association between *NETO1* and schizophrenia remains to be investigated in further studies.

The second caveat is that our study design was based on the common disease, common variant hypothesis, based on which we applied a minor allele frequency threshold (>5%) and selected eight SNPs for follow-up. In the best case scenario, common variants detected in GWAS can explain only part of the heritability in cases of schizophrenia (∼30%) [19] and missense or nonsense mutations on the one side and structural variations (i.e., copy number variants (CNVs)) on the other side are likely to contribute to the increased susceptibility [20]. Recently, the concept of synthetic associations has been suggested, though some there are some objections [21]. Uncommon or rare genetic variants can easily create synthetic associations that are credited to common variants. This possibility requires careful consideration in the interpretation and follow up of GWAS signals [22].

The third caveat in our association study is that cases and controls in replication samples were not matched in age. In other words, although highly unlikely, the controls may develop schizophrenia at some point in life, as they were significantly younger than cases.

The fourth caveat is related to the validity of cognitive function analysis. The premise that *NETO1* was associated with cognitive function in humans had been derived from results of a knock-out mice study [7]. *Neto1*-null mice showed impaired spatial learning measured by the Morris water maze task, the delayed matching-to-place version of the Morris water maze task and displaced-object tasks. In the current study, we investigated executive function (WCST) and vigilance/attention (CPT-IP), however, the results of these cognitive tests might not represent similar cognitive dysfunctions that were shown in the *Neto1*-null mouse study. It may be useful to examine different domains of cognitive impairment associated with *NETO1* in schizophrenic patients using broader cognitive assessment tools.

In conclusion, we were not able to detect evidence for an association between *NETO1* and schizophrenia in the Japanese population. Common variants within the *NETO1* locus may not increase the genetic risk for schizophrenia in the Japanese population. Additionally, common variants investigated in the current study did not affect cognitive performance, as measured by the CPT and the WCST.

## Materials and Methods

### Participants

This study was approved by the Ethics Committees of the Nagoya University Graduate School of Medicine and Fujita Health University, and written informed consent was obtained from each participant. Patients were included in the study if they (1) met DSM-IV criteria for schizophrenia, (2) were physically healthy and (3) had no mood disorders, substance abuse, neurodevelopmental disorders, epilepsy or known mental retardation. A general characterization and psychiatric assessment of subjects is available elsewhere [23]. Controls were selected from the general population. Control subjects had no history of mental disorders, based on questionnaire responses from the subjects themselves during the sample inclusion step, and based on an unstructured diagnostic interview done by an experienced psychiatrist during the blood collection step. The JGWAS sample was comprised of 575 patients with schizophrenia (43.5±14.8 years (mean±s.d.), male 50%) and 564 healthy controls with no personal or family history of psychiatric illness (44.0±14.4 years (mean±s.d.), male 49.8%). All subjects were unrelated, living in the central area of the Honshu island of Japan and self-identified as members of the Japanese population. Subjects of replication samples consisted of 963 schizophrenic patients (47.7±0.5 years (mean±s.d.), male 55.2%) and 919 healthy controls (45.0±0.5 years (mean±s.d.), male 51.0%). The JGWAS and replication samples were collected independently at each university hospital.

### Genotyping and data analysis

Based on the JGWAS results we initially selected SNPs with probability values, p<0.05 and allelic frequencies, MAF>0.05 within the *NETO1* locus. Then we identified redundant SNPs based on the linkage disequilibrium or LD pattern within the interrogated region. Specifically, if the correlation coefficient between two loci (r^2^) was 0.8 or higher, only one of the two loci was selected for the association study [24]. The correlation coefficient between two loci (r^2^) was calculated using Haploview v.4.1 based on the HapMap database (release no. 24, population: Japanese in Tokyo). Finally, we selected seven nonredundant SNPs within the *NETO1* locus. Moreover, one common polymorphism (rs9962470), which showed a low p-value (p = 0.000154) in a previous GWA study [14], was included for genotyping. All eight SNPs are intronic polymorphisms. DNA was extracted from peripheral blood according to a standard protocol [23]. Genotyping was performed using a fluorescence-based allelic discrimination assay (Taqman, Applied Biosystems, Foster City, CA, USA). Power was calculated according to the methods of Skol et al. [25].

To exclude low-quality DNA samples or genotyping probes, data sets were filtered on the basis of SNP genotype call rate (more than 90%) or checked deviation from Hardy-Weinberg equilibrium (HWE) in the control sample. Subjects whose percentage of missing genotypes was >30% or who had evidence of possible DNA contamination were excluded from subsequent analyses.

To reduce the total number of tests, eight associated markers were selected based on the JGWAS results. Next, conditional on the findings of the JGWAS, which used a less stringent nominal level, a meta-analysis was done involving the confirmation sample using the replication data and data from the JGWAS. In the replication sample, Fisher's exact test was used to compare allele frequencies between patients and control subjects. The significance level was set at p<0.05. In the replication sample set, log likelihood ratio tests for assessing haplotype-wise association between schizophrenia and a combination of tagging SNPs was performed using UNPHASED software v3.04. The rare haplotype frequency threshold was set at 5% [26]. In this meta-analysis, p-values were generated by a Cochran–Mantel–Haenszel stratified analysis, and the Breslow–Day test was performed for evaluations of heterogeneous associations as implemented in gPLINK v.2.050 [27].

### Neurocognitive assessment

#### 1. CPT

We used the Continuous Performance Test–Identical Pairs Version Release 4.0 (NewCPT.exe, Copyright 1982–2004 by Barbara A. Cornblatt, All Rights Reserved). The size of PC monitor used for the test was 10.4 inches as each letter was at least 2.2×1.5 cm [28]. Stimuli were flashed on the screen at a constant rate of 1 per second, with a stimulus “on” time of 50 ms. Stimuli were four-digit numbers and were presented 150 times. In each 150-trial condition, 30 of the trials (20%) were target trials and required a response. Target trials were those on which the second of a pair of two identical stimuli appeared [28]. The outcome measure was a mean, d′.

#### 2. WCST

The WCST [29] mainly assesses executive function including cognitive flexibility in response to feedback. We used a modified and computerized version of the test: Wisconsin Card Sorting Test (Keio Version) (KWCST) [30]. The outcome measures were numbers of categories achieved (CA), total errors (TE), and perseverative errors of Milner (PEM) and Nelson types (PEN) in the first trial. We selected outcomes in the WCST, following a prior study, which used KWCST as a measure of cognitive function [31].

(1) CA: This is the number of categories for which six consecutive correct responses are achieved (eight is the maximum number of categories which can be achieved), and is the sum measure of the level of conceptual shifts in the KWCST.

(2) PEN: This is the number of incorrect responses in the same category as the immediately preceding incorrect response (maximum of 47 perseverative errors) [32].

(3) PEM: This is the number of incorrect responses in the same category as the immediately preceding correct response after the category changes.

(4) TE: This is the total number of incorrect responses [33].

#### 3. Clinical information

Chlorpromazine (CPZ) equivalent doses were calculated based on the report by Inada [34], [35]. The Positive and Negative Symptom Scale (PANSS) was used to evaluate patients [36].

#### 4. Analysis of cognitive performance

From the sample used in the current study, we made a subset of randomly selected participants older than 18 years of age for analysis of cognitive performance. Cognitive data analysis was done for the participants who completed both WCST and CPT-IP. We checked the effect of three SNPs on cognitive performance measured by the Continuous Performance Test and the Wisconsin Card Sorting Test (107 schizophrenic patients, 104 Healthy controls). IBM SPSS statistical software, version 19 was used for all analyses. We compared age, education, CPZ equivalent doses, age at onset, duration of illness, positive scale, negative scale and General Psychopathology Scale between schizophrenia cases and control subjects using a two-tailed t-test and Welch's t-test. We compared sex between case and control groups using Fisher's exact test. Next, we compared d′ in the CPT and CA, PEM, PEN, TE in the WCST between the case and control groups using a two-tailed t-test and Welch's test (Table S4).

## Supporting Information

Method S1 Meta-analysis.
(DOC)

Table S1 Haplotype analysis of the eight SNPs of *NETO1*.
(DOC)

Table S2 Allele frequencies of the eight SNPs of *NETO1* in males.
(DOC)

Table S3 Allele frequencies of the eight SNPs of *NETO1* in females.
(DOC)

Table S4 Cognitive performance of three SNPs in *NETO1*.
(DOC)
