# Supplementary material for: A Case Control Association Study and Cognitive Function Analysis of Neuropilin and Tolloid-Like 1 Gene and Schizophrenia in the Japanese Population
Source: PLoS One. 2011 Dec 20;6(12):e28929. doi: 10.1371/journal.pone.0028929 (PMC3243668; doi:10.1371/journal.pone.0028929)
Supplement: Method S1 — Meta-analysis. (DOC) [file pone.0028929.s001.doc]

Method S1. Meta-analysis

We identified one SNP (rs6566674) that was included both in our replication sample set and another Japanese GWAS . Their study covered only rs6566674 of *NETO1* in their first stage because their platform was Affymetrix GeneChip Mapping 100K and rs6566674 was not significantly associated with schizophrenia in their first stage using the trio sample set in the transmission disequilibrium test (TDT). Two study designs are commonly employed in genetic association studies: a case-control and a family-based approach. The case-control design compares frequencies of alleles carried among cases with a disease and among controls that are free of disease. The family-based design compares the frequency of alleles transmitted to an affected offspring by their parents with alleles carried by the parents but not passed to the offspring; this type of statistical analysis is often called a TDT . Using the genotype data from both studies, we performed meta-analysis of rs6566674. We investigated whether T allele in rs6566674, which was risk allele in JGWAS, was associated with schizophrenia or not.

We conducted a combined family-based and case-control meta-analysis using the R language case-control and transmission disequilibrium test meta-analysis package (Catmap) . This method implements the Kazeem and Farrell fixed effects and DerSimonian and Laird random-effects estimates of the pooled odds ratio.

The OR was 1.01 (95% confidence intervals: 0.85-1.20) and the P value was 0.90 in fixed effects estimates. The OR was 1.03 (95% confidence intervals: 0.81-1.31) and the P value was 0.80 in random effects estimates. The Q statistic (Heterogeneity) P was 0.19. Therefore, this meta-analysis did not show SNP rs6566674 to be significantly associated with schizophrenia either in fixed or in random effects estimates.

References

1. Yamada K, Iwayama Y, Hattori E, Iwamoto K, Toyota T, et al. (2011) Genome-wide association study of schizophrenia in Japanese population. PloS one 6: e20468.

2. Nicodemus KK (2008) Catmap: case-control and TDT meta-analysis package. BMC bioinformatics 9: 130.

3. Kazeem GR, Farrall M (2005) Integrating case-control and TDT studies. Annals of human genetics 69: 329-335.

4. DerSimonian R, Laird N (1986) Meta-analysis in clinical trials. Controlled clinical trials 7: 177-188.
