# Supplementary material for: A Case Control Association Study and Cognitive Function Analysis of Neuropilin and Tolloid-Like 1 Gene and Schizophrenia in the Japanese Population
Source: PLoS One. 2011 Dec 20;6(12):e28929. doi: 10.1371/journal.pone.0028929 (PMC3243668; doi:10.1371/journal.pone.0028929)
Supplement: Table S2 — Allele frequencies of the eight SNPs of NETO1 in males. (DOC) [file pone.0028929.s003.doc]

Table S2. Allele frequencies of the eight SNPs of *NETO1* in male

^a^ based on NCBI 36

^b^ minor allele frequency

^c^ Fisher's exact test

^d^ Lower (L) and upper (U) 95% confidence intervals

^e^ p-value of Breslow-day test
