# Supplementary material for: A Case Control Association Study and Cognitive Function Analysis of Neuropilin and Tolloid-Like 1 Gene and Schizophrenia in the Japanese Population
Source: PLoS One. 2011 Dec 20;6(12):e28929. doi: 10.1371/journal.pone.0028929 (PMC3243668; doi:10.1371/journal.pone.0028929)
Supplement: Table S4 — Cognitive performance of three SNPs in NETO1. (DOC) [file pone.0028929.s005.doc]

Table S4. Cognitive performance of three SNPs in *NETO1*

^a^ t-test (unless noted otherwise)

^b^ Fisher's exact test

^c^ Welch's t test
